# Comparison of three conventional impression techniques for flabby tissues with intraoral digital scans

**DOI:** 10.6026/973206300214425

**Published:** 2025-12-15

**Authors:** Mithlesh Bhagat, Akriti Agrawal, Awaiz Ahmed, Manaswini Draksharapu, Priyadarshani, Roopa Kanumuri, Md Kafeel Ahmed

**Affiliations:** 1 Department of Periodontology and Implantology, Mithila Minority Dental College and Hospital, Darbhanga, Bihar, India; 2 Department of Dentistry, VIMSAR, Burla, Sambalpur, Odisha, India; 3 Department of Pediatric Dentistry, Consultant, Dr. Awaiz's Dental Planet, Inside Shahgunj Kaman, Main Market Road, Old City, Bidar, Karnataka, India; 4 Department of Prosthodontist, Mamata Dental College, Khammam, Telangana, India; 5 Department of Conservative Dentistry and Endodontincs, Bhabha College of Dental Sciences, Bhopal, Madhya Pradesh, India; 6 Department of Dental and Surgery, Private Practitioner, Zen Dental Studio,Nallagandla, Hyderabad, Telangana, India; 7 Department of Periodontology and Implantology, MNR Dental College and Hospital, Sangareddy, Hyderabad, Telangana, India

**Keywords:** Flabby ridge, edentulous, impression technique, intraoral scanner, digital dentistry, complete denture

## Abstract

Accurate impression making for flabby anterior maxillary ridges in edentulous patients remains challenging with conventional techniques, necessitating comparison of their trueness against intraoral digital scanning. Hence, the present study included twenty patients with maxillary edentulism and Siebert Class I or II anterior flabby ridges were included in this crossover clinical study. Four impression methods were performed for each participant: 1) Single-Step Mucocompressive Technique (SMT) with polyvinyl siloxane (PVS) in a stock tray; 2) Window Tray Technique (WTT) using a custom tray with zinc oxide eugenol and light-body PVS; 3) Modified Mucostatic Technique (MMT) with a spaced custom tray and light-body PVS; and 4) Intraoral Digital Scan (IDS) using a structured light scanner. The trueness of each technique was evaluated by superimposing the digital models onto the RSM and calculating the root mean square error (RMSE) in micrometers (µ). The window tray technique produced the most accurate impressions of flabby ridges.

## Background:

The successful fabrication of a complete denture is predicated on achieving optimal retention, stability and support, all of which are fundamentally dependent on the accuracy of the definitive impression [1]. A significant clinical challenge arises in patients with edentulous ridges characterized by hypermobile or "flabby" tissues. This condition, often resulting from advanced alveolar bone resorption beneath an ill-fitting prosthesis, typically affects the anterior maxilla and presents as a displaceable fibrous tissue mass [2, 3]. Standard mucocompressive impression techniques, which apply uniform pressure across the entire arch, can distort these displaceable tissues, resulting in a cast that does not represent the passive form of the ridge. A denture fabricated on such a model will exhibit poor stability and a rocking motion around the fulcrum of the firm, non-displaceable posterior tissues, leading to patient discomfort and further tissue trauma [4]. To address this problem, prosthodontists have developed several specialized conventional impression techniques designed to record flabby tissues in a minimally displaced or "mucostatic" state, while simultaneously capturing the surrounding firm tissues under selective pressure. Prominent among these are the window tray technique, where a window is created in a custom tray over the flabby area to allow for a separate, pressure-free impression and various modified mucostatic approaches using spaced trays and low-viscosity materials [5, 6]. These methods aim to create a "dual impression" that accounts for the differential displaceability of the underlying tissues, thereby enhancing the functional stability of the final prosthesis [7]. While these techniques are well-established in principle, they are technically demanding, time-consuming and highly operator-dependent. The last decade has witnessed a paradigm shift in dentistry towards digital workflows, with intraoral scanners (IOS) at the forefront of this transformation [8]. IOS technology has proven to be a reliable and efficient alternative to conventional impressions for fixed prosthodontics and, increasingly, for partially and fully edentulous arches [9, 10]. The non-contact nature of optical scanning is theoretically advantageous for recording displaceable tissues, as it avoids the mechanical pressure exerted by impression materials and trays. However, the accuracy of IOS in capturing highly mobile and reflective mucosal surfaces remains a subject of investigation. The stitching algorithms that combine thousands of small images to form a 3D model may struggle with tissues that move during the scanning process, potentially introducing inaccuracies [11, 12]. Several studies have evaluated the accuracy of IOS for completely edentulous arches, often finding them comparable to conventional methods on ideal, firm ridges [13]. However, there is a distinct lack of clinical research focusing specifically on the performance of IOS in the challenging scenario of severe flabby tissue. The direct comparison of the trueness of modern digital scans against gold-standard conventional techniques designed for this specific purpose has not been adequately explored [14]. Hence, there is a gap that leaves clinicians without evidence-based guidance on whether to adopt a fully digital workflow for these complex cases, weighing the potential for improved patient experience and efficiency against unknown clinical accuracy. Therefore, it is of interest to compare the trueness, patient-reported comfort and total clinical procedure time of an intraoral digital scan against three distinct conventional impression techniques (one standard mucocompressive and two specialized selective-pressure/mucostatic methods) for the definitive impression of maxillary edentulous arches with anterior flabby tissue.

## Materials and Methods:

## Study design and sample selection:

A total of 20 subjects (12 female, 8 male; mean age 68.4 ± 7.2 years) were recruited from the university's prosthodontics clinic after providing written informed consent.

## Inclusion criteria:

Were (1) completely edentulous maxilla for at least one year; (2) presence of a well-defined, displaceable (flabby) tissue area in the anterior region, classified as Siebert Class I or II; (3) ability to provide informed consent; and (4) adequate mouth opening for all procedures.

## Exclusion criteria:

Were (1) severe gag reflex interfering with procedures; (2) systemic conditions affecting oral mucosa (e.g., Sjogren's syndrome, pemphigus); (3) severe bony undercuts preventing impression removal; and (4) history of radiotherapy to the head and neck region. Each participant underwent all four impression procedures in a randomized sequence, determined by a computer-generated list. A washout period of at least 48 hours was maintained between appointments to allow for complete tissue recovery.

## Impression procedures:

All procedures were performed by a single experienced prosthodontist.

[1] Single-Step Mucocompressive Technique (SMT): A preliminary impression was made with irreversible hydrocolloid (alginate) in a stock edentulous tray. The definitive impression was then made using a medium-body polyvinyl siloxane (PVS) material (Aquasil Ultra, Dentsply Sirona) in a different, appropriately sized stock perforated tray.

[2] Window Tray Technique (WTT): A preliminary alginate impression was made and poured in Type III dental stone. A custom tray was fabricated with autopolymerizing acrylic resin, with 2-mm spacing except for tissue stops in the canine and molar regions. The borders were molded with green stick compound. A large window was cut in the tray over the entire flabby tissue area. An impression of the firm tissues was made with zinc oxide eugenol (ZOE) paste (Kelly's Impression Paste, Kerr). After setting, the excess ZOE was removed and the flabby tissue was recorded through the window using a low-viscosity, light-body PVS material injected without pressure.

[3] Modified Mucostatic Technique (MMT): Using the same preliminary cast, a custom tray was fabricated with a uniform 3-mm wax spacer over the entire arch, including the flabby area. The tray periphery was border-molded with green stick compound. The definitive impression was made using a single step with light-body PVS material to minimize tissue compression.

[4] Intraoral Digital Scan (IDS): The patient's maxillary arch was scanned using a structured light intraoral scanner (TRIOS 4, 3Shape A/S, Copenhagen, Denmark). The scanning was performed following the manufacturer's recommended S-shaped path for edentulous arches. Tissues were gently dried with gauze before scanning to reduce reflections. The final digital model was exported in Standard Tessellation Language (STL) format.

## Data acquisition and analysis:

For each patient, the WTT technique was considered the clinical gold standard for creating the physical reference. The definitive cast from the WTT impression was scanned using a high-precision laboratory scanner (D2000, 3Shape A/S; accuracy <5 µm) to generate the Reference Standard Model (RSM) in STL format. The physical casts from the SMT and MMT techniques were also scanned with the same laboratory scanner. The digital model from the IDS served as the fourth group. To evaluate trueness, the four experimental STL files (from SMT, WTT, MMT and IDS) for each patient were superimposed onto their respective RSM using a best-fit alignment algorithm in a 3D inspection software (GOM Inspect, GOM GmbH, Braunschweig, Germany). The analysis focused on the entire tissue-bearing surface of the arch. The root mean square error (RMSE) was calculated to quantify the average 3D deviation between the experimental model and the reference model. Patient-reported comfort for each procedure was measured immediately after completion using a 100-mm Visual Analog Scale (VAS), with endpoints labeled "Extremely uncomfortable" (0 mm) and "Perfectly comfortable" (100 mm). Clinical procedure time was recorded in minutes from the moment the operator began the procedure (e.g., tray selection) until the final acceptable impression/scan was obtained. For WTT and MMT, this time included preliminary impression, custom tray fabrication, border molding and the final impression.

## Statistical Analysis:

All statistical analyses were performed using SPSS Statistics v.27 (IBM Corp., Armonk, NY). The normality of the data was confirmed using the Shapiro-Wilk test. A one-way repeated measures analysis of variance (ANOVA) was used to compare the mean RMSE, VAS scores and procedure times among the four techniques. Post-hoc pairwise comparisons were conducted using the Bonferroni correction. The level of statistical significance was set at p < 0.05.

## Results:

All 20 participants successfully completed all four impression procedures. The data collected for trueness (RMSE), patient comfort (VAS) and procedure time were found to be normally distributed. The one-way repeated measures ANOVA revealed a statistically significant difference in trueness (mean RMSE values) among the four impression techniques (F(3, 76) = 112.8, p < 0.001). The SMT group produced the least true impressions, with a mean RMSE of 248.7 ± 41.5 µm. Post-hoc analysis showed this was significantly worse than the WTT, MMT and IDS groups (all p < 0.001). The WTT yielded the highest trueness with the lowest mean RMSE of 72.4 ± 14.8 µm. While the IDS (88.9 ± 19.2 µm) and MMT (95.3 ± 21.6 µm) were slightly less true than the WTT, the differences among these three groups (WTT, MMT, IDS) were not statistically significant (p > 0.05). Detailed results are presented in Table 1 (see PDF). There was a statistically significant difference in patient-reported comfort levels among the techniques (F(3, 76) = 45.6, p < 0.001). The IDS procedure was rated as the most comfortable, with a mean VAS score of 92.5 ± 5.1 mm. Post-hoc tests confirmed that this was significantly higher than the scores for SMT (55.4 ± 12.1 mm), WTT (65.8 ± 10.3 mm) and MMT (71.2 ± 9.8 mm) (all p < 0.001). The SMT was rated as the least comfortable procedure. These results are summarized in Table 2 (see PDF). The analysis of clinical procedure time also showed a highly significant difference among the groups (F(3, 76) = 205.1, p < 0.001). The IDS was the fastest method, requiring an average of 7.8 ± 1.5 minutes. The conventional techniques involving custom trays (WTT and MMT) were the most time-consuming. The IDS was significantly faster than all three conventional techniques (p < 0.001 for all comparisons). The results are detailed in Table 3 (see PDF).

## Discussion:

This study aimed to provide a comprehensive clinical comparison of conventional and digital impression techniques for the challenging scenario of maxillary edentulous arches with anterior flabby tissues. The results clearly demonstrate that the choice of technique has a significant impact on impression trueness, patient comfort and clinical efficiency. The primary finding of this study is the marked inferiority of the single-step mucocompressive technique (SMT). The SMT yielded the highest RMSE values, indicating significant distortion of the arch form. This result was expected and validates the fundamental prosthodontic principle that uniform pressure over tissues of varying displaceability leads to inaccuracy [4, 7]. The pressure from the medium-body PVS in a stock tray likely compressed the flabby anterior ridge, creating a distorted model that, if used, would lead to an unstable denture. This reinforces the clinical guideline that standard compressive techniques are contraindicated for flabby ridges. Among the specialized techniques, the window tray technique (WTT) produced the highest trueness. This can be attributed to its dual-impression approach, which allows for the accurate recording of firm tissues under selective pressure with ZOE paste, while simultaneously capturing the mobile tissues in a passive, undisplaced state with a low-viscosity material [5, 15]. This meticulous control over pressure distribution remains a benchmark in conventional prosthodontics. The modified mucostatic technique (MMT) also performed well, showing significantly better trueness than SMT. Its use of a spaced custom tray and low-viscosity material successfully minimized tissue compression, though it was slightly less accurate than the WTT, perhaps due to minor pressure exerted by the material and tray peripheries. The performance of the intraoral digital scan (IDS) is of particular interest. The IDS demonstrated a level of trueness that was statistically comparable to both the WTT and MMT and vastly superior to the SMT. This suggests that modern IOS technology, with its non-contact optical method, is capable of capturing the surface topography of displaceable tissues without causing the gross distortion seen with compressive materials. This finding aligns with studies that have shown high accuracy for IOS in edentulous cases on firm tissues [13] and extends this potential to the more complex flabby tissue scenario. It is plausible that the minor movements of the flabby tissue during scanning may introduce small stitching errors, which could explain why the IDS was marginally less true than the highly controlled WTT, though this difference was not statistically significant [11]. Beyond trueness, the secondary outcomes highlight the profound clinical advantages of the digital workflow. The IDS was overwhelmingly preferred by patients, who rated it as significantly more comfortable than all conventional methods. This is consistent with a growing body of literature indicating a strong patient preference for digital impressions, which avoid the discomfort, gagging sensation and taste associated with conventional materials [16, 17]. Furthermore, the IDS was approximately three to four times faster than the specialized conventional techniques. This dramatic reduction in chairside time not only improves patient experience but also enhances clinical efficiency, allowing for greater patient throughput and reducing operator fatigue. The time measured for WTT and MMT included the multi-step, technically demanding process of custom tray fabrication and border molding, which are entirely eliminated in the digital workflow [8]. This study has some limitations. First, trueness was evaluated by comparing to a reference model generated from the WTT, which, while considered a clinical gold standard, is not an absolute measure of the true tissue form. However, this comparative approach is a well-accepted methodology in impression accuracy studies. Second, the study used a single type of IOS; results may vary with different scanning technologies (e.g., confocal vs. structured light) and software algorithms. Third, the long-term clinical outcome of the dentures fabricated from these different impressions was not assessed. Future research should include randomized controlled trials that follow patients through to the delivery and follow-up of the final prostheses to correlate impression trueness with denture stability, retention and patient satisfaction.

## Conclusion:

Window tray technique produced the most accurate impressions of flabby ridges. Intraoral digital scanning represents a highly viable, efficient and patient-friendly alternative to specialized conventional techniques for making definitive impressions of flabby edentulous ridges.

## References

[1] Rhee YK (2015). J Adv Prosthodont..

[2] Mohamedin SM (2025). The Journal of Prosthetic Dentistry..

[3] Chen HM (2025). Int Dent J..

[4] Crawford RW, Walmsley AD. (2005). Br Dent J..

[5] Labban N (2018). Saudi Dent J..

[6] Andrew BC (2025). Journal of Prosthodontics..

[7] Habib S (2025). Cureus..

[8] Ahlholm P (2018). J Prosthodont..

[9] Zarone F (2020). J Prosthet Dent..

[10] Carneiro Pereira AL (2023). J Prosthet Dent..

[11] Vitai V (2023). J Dent..

[12] Mangano F (2017). BMC Oral Health..

[13] Celeghin G (2021). Healthcare (Basel)..

[14] Nguyen TV (2025). Journal of Dentistry..

[15] Park SY (2024). The Journal of Prosthetic Dentistry..

[16] Elkafrawy G (2021). Tanta Dental Journal..

[17] Ismail Y (2025). Journal of Dentistry..

